# The silent giant

**DOI:** 10.11604/pamj.2021.38.147.28030

**Published:** 2021-02-09

**Authors:** Sofia Pina, Susana Rodrigues

**Affiliations:** 1 Serviço Cirurgia Geral, Centro Hospitalar Universitário Lisboa Central, Sofia Pina, Lisbon, Portugal

**Keywords:** Gallbladder, giant, adenocarcinoma

## Image in medicine

A 61-year-old male, with history of cardiovascular disease, entered the Emergency Department due to jaundice, pruritus and acholia with 1 week evolution. The physical examination revealed a large mass in the right hypochondrium, with no pain or defense on palpation. Analytically, there was no increase in inflammatory parameters, but total bilirubin value was 14.9mg/dL, direct bilirubin 8.0mg/dL and there was a slight increase in transaminases (AST 99U/L and ALT 64U/L) along with an increase in GGT (472U/L). He was submitted to an abdominal computed tomography (CT) scan that revealed a large liquid mass (189mm of larger diameter) that extended through the right hypochondrium and right flank, with a cranial pole at the level of the hepatic hilum. This was causing dilation of the hepatic channels and intrahepatic bile ducts as well as compression of the nearby structures (pancreatic head). Due to obstructive jaundice, the patient was admitted and went into surgery. Intraoperatively, we saw a gallbladder with approximately 20cm long axis. He was submitted to a cholecystectomy with resection of the main bile duct and Roux-en-Y hepaticojejunostomy. In the postoperative period there was the need for a prolonged admission in the intensive care unit (ICU) due to septic shock with multiorgan dysfunction but with progressive recovery. The patient was discharged after 45 days of hospitalization. The anatomy of the specimen revealed an adenocarcinoma involving the main bile duct and the gallbladder, G1, pT3Nx stage.

**Figure 1:**
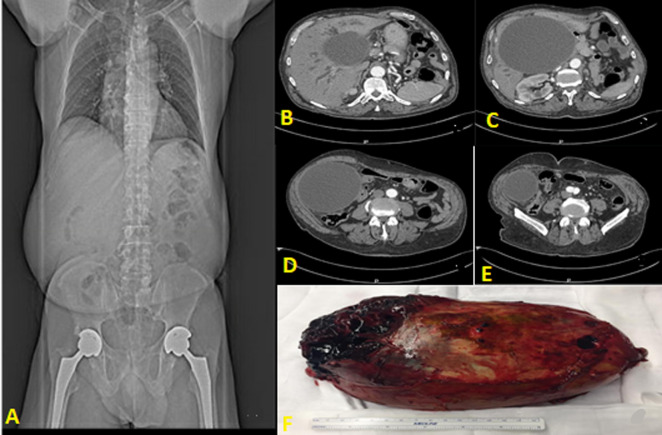
gallbladder-CT images and surgical specimen

